# Otorhinolaryngology Symptom Assessment Using SNOT 22 Among SARS CoV-2 Patients in a Tertiary Care Centre

**DOI:** 10.1007/s12070-022-03340-6

**Published:** 2022-12-19

**Authors:** Sanchit Bajpai, Rakshitha Samanth, Vijendra Shenoy, Kshithi Kudlu, Sushmitha Bhat, Farnaz Nasrin Islam, Sreenivas Kamath Kasargod

**Affiliations:** Department of Otorhinolaryngology and Head and Neck Surgery, Kasturba Medical College, Mangaluru, Manipal Academy of Higher Education, Manipal, Karnataka India

**Keywords:** ENT symptoms, COVID-19, SNOT-22

## Abstract

The main aim of the study was to assess various ENT symptoms in COVID 19 patients, also to investigate the severity of ENT symptoms among COVID 19 patients and find their relation on basis of scores among five discrete domains of SNOT 22 (Sino nasal Outcome Test). A prospective observational study was conducted among 135 patients between 18 to 75 years of age, in the month of September 2020 with COVID-19 infection having mild, moderate disease who were admitted to our hospital. Subjects were divided into groups according to their presenting ENT symptoms based on age, gender and other comorbidities and differences between the groups were examined. The sinonasal symptoms were assessed using the SNOT 22 questionnaire. A strong statistical significance with loss of smell and taste sensation was noted in patients above the age of 40 years. It was also noted that the patients who presented with cough above the age of 40 years were significantly more. Evaluation of sinonasal symptoms using SNOT 22 questionnaire showed that Extranasal rhinologic symptoms, Psychological dysfunction, Sleep dysfunction had significantly higher association among patients who were more than 40 years. We observed that, Extranasal rhinologic symptoms were significantly higher among males than females. There is thus an emergent need to develop a uniform tool to assess the various ENT symptoms. In our study we assessed the patients with COVID 19 using a standard questionnaire to observe the symptomatology, psychological and sleep dysfunctions due to sinonasal issues, and to closely understand the relationship of various symptoms in a meticulous manner.

## Introduction

The coronavirus disease 2019 (COVID-19) is a continuing viral pandemic that emerged from East part of Asia and rapidly disseminated to the other parts of the world. It has been observed that the transmission between the humans has led to an exponential rise in many areas of the world.

COVID-19 manifestations involves a broad clinical spectrum ranging from asymptomatic features to multi-organ failures [1]. Despite its quick spread worldwide, the clinical manifestations of COVID-19 mostly remain obscure to a larger extent. The nasopharyngeal and/or the oropharyngeal tissue is one of the main nurture sites of the infection and the key site of taking the swab for sample testing [2]. Various clinical studies showed that the most prevalent symptoms of novel corona virus among patients are fever, dry cough, shortness of breath, generalized myalgia, joint pain, headache, diarrhea, nasal discharge, and throat irritation [3]. However, most published literatures on COVID-19 concentrated on the lower respiratory tract clinical characteristics and sequels due to their lethal nature. The clinical course of the disease has shown subtle signs of upper respiratory symptoms which act as an important indicator, helping the clinician in early diagnosis and halt the further progression of the disease. The studies on Otorhinolaryngological manifestations in COVID-19 infection is limited hence, there is a great need in understanding pattern of ENT symptoms of this novel virus and there is a considerable requirement to recognize the defining ENT clinical manifestations with more accuracy. To the best of our attention, there is no previous study to outline the ENT symptoms in COVID-19 patients with their Quality of life assessment.

Thus, the aim of the present work was to detect and discuss the different otorhinolaryngological symptoms in patients with laboratory confirmed COVID-19. The goal of the current study was also to investigate the severity of ENT symptoms among COVID-19 patients and find their relation on basis of scores among five discrete domains of SNOT 22 (Sino nasal Outcome Test) [4].

## Materials and Methods

An observational study was conducted over a period of 1 month at a tertiary referral center. The protocol was approved by the Institutional Ethics Committee (No.: IECKMCMLR-08/2020/237). One hundred thirty-five individuals with lab confirmed cases who were over 18 years old, suffering from mild to moderate instances, and who were hospitalized were included in our study. The inclusion criteria were being Above 18 years of age of both gender with laboratory-confirmed COVID-19 infection with mild and, moderate infection. Asymptomatic and severe COVID infection, not willing to participate, chronic rhinosinusitis, prior ENT surgeries, sinonasal malignancy, head and neck trauma, previous radiation to head and neck, degenerative neurological disease, any mental health disorders were excluded from the study. Informed consent was obtained from all patients before participating in the study.

All patients included in our study had positive results of either COVID-19 RTPCR test or Rapid Antigen Test, which was studied with samples of nasopharyngeal/ oropharyngeal swab. All patients who fulfilled the above criteria’s and had agreed to participate in the study were provided a google form having subject information, consent form and demographic and basic medical information in the proforma along with SNOT 22 questionnaire using the google form link.

All the participants were asked to fill the SNOT 22 questionnaire. SNOT 22 is a validated standard 22 item tool applicable to sinonasal conditions. High scores on the SNOT 22 items are suggestive of worsened patient symptom severity (Total score 0–110). The scoring is conducted by Likert scale responses where 0 = No problem; 1 = Very mild problem; 3 = Mild/slight problem; 4 = Severe problem; 5 = Problem as bad as it can be. The 22 items of the SNOT 22 scores were divided into 5 distinct domains which were Rhinologic symptoms, Extra-nasal rhinologic symptoms, Ear/ facial symptoms, Psychological dysfunction, Sleep dysfunction.

Statistical analysis was performed using Statistical package SPSS version 25.0 Categorical variables were presented as frequency and percentage. Continuous variables was described as median (Q1, Q3). Chi square test was performed to see the association between symptoms versus age, gender, concomitant diseases. Mann–Whitney U test was carried out to compare median values of different domains of SNOT 22 between the age and gender category. *P* value < 0.05 was considered as significant.

## Results

One hundred and thirty-five participants were included in our study, out of which 66 were males and 69 were females. All participants had to answer a google based questionnaire which included specific ENT symptoms and their severity were assessed using SNOT 22 Tool. The patients ranged in age from 18 to 75 years.

**Fig. 1:**
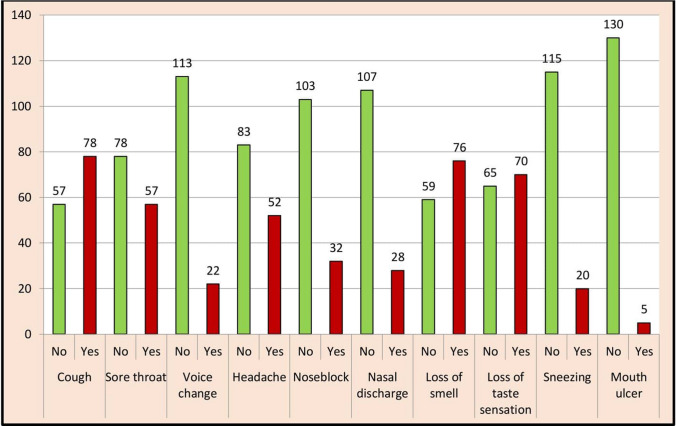
Distribution of frequency of otorhinolaryngological symptoms

We observed the most prevalent comorbidities in these patients were hypertension (7.4%), diabetes (6.7%) as depicted in Table 1. The severity classification of COVID compared with age is depicted in Table 2 which was found to be highly significant (*P* < 0.001). The particulars regarding the frequency of ENT symptoms has been depicted in Fig. 1. The most common symptoms were cough, loss of smell and loss of taste, sore throat respectively.

**Table 1:** Demographic data

Variable	N (%)
Age ≤ 40 years > 40 years	80 (59.3) 55 (40.7)
Gender Male Female	66 (48.9) 69 (51.1)
Co-morbidities No Allergic rhinitis Asthma Hypertension Hypertension and cardiovascular disease Diabetes Diabetes and hypertension Cardiovascular disease Diabetes, hypertension and asthma Asthma and allergic rhinitis Diabetes and cardiovascular disease	100 (74.1) 3 (2.2) 2 (1.5) 10 (7.4) 1 (0.7) 9 (6.7) 4 (3) 2 (1.5) 2 (1.5) 1 (0.7) 1 (0.7)

**Table 2:** The severity classification of COVID compared with age

Age category	Severity		Chi square	*P* value
	Mild	Moderate		
≤ 40 years	62 (78.5)	17 (21.5)	18.79	< 0.001
> 40 years	23 (41.8)	32 (58.2)		

In our study, detailed ENT related clinical symptoms were accounted among all 135 patients included. Patients were subgrouped according to age and gender as shown in Table 3. It was interesting to note that the cases included in our study showed a strong statistical significance with loss of smell and taste sensation (*P* < 0.001). The highlight finding in our study was that the number of patients having loss of smell and taste sensation were singnificantly more above the age of 40 years. It was also noted that the patients who presented with cough above the age of 40 years were significantly more (*P*-0.01) as shown in Table 3.

**Table 3:** Assessment of distribution of patients with their symptoms as per age and gender

Categories symptoms	Age		*P*	Gender		*P*
	≤ 40 years	> 40 years		Male	Female	
Cough No Yes	41 39	16 39	0.01	23 43	34 35	0.09
Sore throat No Yes	50 30	28 27	0.18	33 33	45 24	0.07
Voice change No Yes	68 12	45 10	0.62	56 10	57 12	0.72
Headache No Yes	50 30	33 22	0.76	44 22	39 30	0.22
Nose block No Yes	61 19	42 13	0.98	55 11	48 21	0.06
Loss of smell No Yes	45 35	14 41	< 0.001	24 42	35 34	0.09
Loss of taste sensation No Yes	50 30	15 40	< 0.001	31 35	34 35	0.78
Sneezing No Yes	69 11	46 9	0.67	59 7	56 13	0.17
Running nose/Nasal discharge No Yes	62 18	45 10	0.54	53 13	54 15	0.77
Mouth ulcer No Yes	76 4	54 1	0.64	63 3	67 2	0.67

**Fig. 2:**
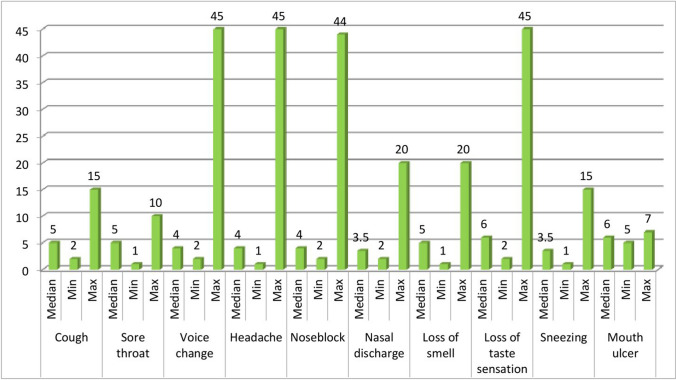
Data on the maximum, minimum and median duration of the symptoms

Data on the minimum, maximum and median duration of the symptoms were observed. Accordingly, the longest-onset ENT symptoms were observed to be Voice change, headache, loss of taste sensation and nasal block respectively. There was no significant difference between the other symptom duration averages (Fig. 2).

Mann–Whitney U test was done to compare the median values of different domains of SNOT 22 questionnaire between age groups and gender. It was noted that Extranasal rhinologic symptoms (*P* = 0.005), Psychological dysfunction (*P* = 0.03) and Sleep dysfunction (*P* = 0.002) had significantly higher association among patients more than 40 years (Table 4). It was also seen that Extranasal rhinologic symptoms were significantly higher among males (*P* = 0.01) than females (Table 5).

**Table 4:** Comparing the median values of different domains of SNOT 22 questionnaire between age groups

Variable	Age		Mann–Whitney	*P*
	≤ 40 Years median (Q_1_, Q_3_)	> 40 Years median (Q_1_, Q_3_)		
Rhinologic symptoms	1 (0, 4)	1 (0, 4)	2056	0.50
Extranasal rhinologic symptoms	1 (0, 3)	3 (2, 4)	1581	0.005
Ear/facial symptoms	0 (0, 2)	0 (0, 2)	2146.5	0.78
Psychological dysfunction	2 (0, 9)	5 (2, 8)	1722.5	0.03
Sleep dysfunction	1 (0, 4)	3 (2, 6)	1525	0.002

**Table 5:** Comparing the median values of different domains of SNOT 22 questionnaire between gender

Variable	Gender		Mann–Whitney	*P*
	Male median (Q_1_, Q_3_)	Female median (Q_1_, Q_3_)		
Rhinologic symptoms	1 (0, 3.25)	2 (0, 4)	1941	0.12
Extranasal rhinologic symptoms	3 (0.75, 4)	2 (0, 3)	1756	0.01
Ear/facial symptoms	0 (0, 2)	0 (0, 2)	2052	0.26
Psychological dysfunction	3 (1.75, 7)	4 (0, 9.5)	2146.5	0.56
Sleep dysfunction	2 (0.75, 5)	2 (0, 5)	2231	0.83

## Discussion

COVID-19 is a rapidly spreading disease which originated in Wuhan in the late 2019 and currently spread across the world incredibly quickly, killing millions of people in its wake. The principle route of spread of SARS-Cov-2 is through droplets and also by touching droplet contaminated hands to regions of the face such as oral cavity, nostrils, or eyes. Hence, it is obvious that patients will present with upper respiratory tract infections, since it is the principal entry point into the body [5]. Knowledge regarding the natural history and initial symptoms will aid in revealing COVID-19 disease further, and set up additional effectual management protocols.

Symptoms at the beginning of COVID-19 infection in admitted patients in China were fever, cough dry or productive, fatigue, bodyache, breathlessness, chest discomfort, loose stools, headache, anorexia, throat irritation, giddiness, palpitations and vomiting [6]. Study conducted by Elif Elibol, depicted that the highest portion of ENT symptom was cough which was seen in 43.8%, and sore throat, and loss of smell (35.4%). They concluded that most significant predictive ENT symptoms were cough, loss of smell sensation, and sore throat [7]. In the study done by Chaurasia et al., throat symptoms were found to be the most common symptoms including taste dysfunction in 74 patients out of 465 patients [8]. Systematic review conducted by Joanna Krajewska1 et al. found that the most prevalent ENT symptoms seen in patients with SARS-CoV-2 were dry cough, sore throat and breathing difficulty [9]. In our study, we found that out of 135 patients, most common Otolaryngological symptom was cough which was seen among 78 patients, followed by loss of smell, loss of taste and sorethroat.

Sinonasal symptoms such as nose block and nasal discharge were noted in 32 (23.7%) and 28 (20.7%) patients respectively, which did not correspond with the olfactory dysfunction 76 (56%) patients. Similar findings were noted in study conducted by Maryam Jalessi et al. [10]. No correlation identified between the sinonasal involvement and olfactory dysfunction in our patients. This proves that the olfactory dysfunction is not due to edema of the nasal mucosal lining and/or obstruction of the nasal cavity, which usually occurs during sinonasal infections. ACE2 is recognized as the functional receptor for SARS CoV-2, which is present in various organs of our body, including skeletal muscle and nervous system. The expression and distribution of this receptor proves that the SARS CoV-2 can cause neurologic phenomenon via direct or indirect processes [11]. It is evident that SARS-CoV-2 can travel across the lamina of the cribriform plate of ethmoid through the ACE2 receptor, and it is through the olfactory nerve, transneuronal transportation is attained, which can later lead to severe neurological symptoms such as altered consciousness, acute cerebrovascular event, meningitis, encephalitis, ataxia, and seizure. Patients may present with only nonspecific neurologic symptoms such as anosmia or aguesia, headache, myalgia as their presenting symptoms. Therefore among COVID 19 patients, neurologic symptoms should be closely monitored.

The incidence of olfactory and gustatory dysfunction is higher in individuals more than 40 years of age. There was no significant difference between association of loss of smell and taste with gender. Most of the previous studies have shown that the incidence of smell disorders in patients with COVID-19 is more in females compared to males [12]. A systematic review and meta-analysis done by Akosua Adom Agyeman et al. revealed that Increasing age related with lower prevalence of olfactory and taste dysfunctions, whereas the use of objective methods of assessment related with higher prevalence of olfactory dysfunction. No significant difference was found between the prevalence of olfactory, gustatory dysfunctions and sex [13].

SNOT 22 Questionnaire is one of the tools that uncovers most of the physical issues, functional limitations and also the emotional sequelae of patients suffering from sinonasal disease [14]. We used this questionnaire in our study since most of the symptoms of COVID-19 are uncovered in this questionnaire which can aid us in determining the Quality of life in patients suffering from COVID-19 infection, we found that Extranasal rhinologic symptoms, Psychological dysfunction and Sleep dysfunction had significantly higher association among patients more than 40 years (Table 4). Also Extranasal rhinologic symptoms were significantly higher among males (*P* = 0.01) than females (Table 5) indicating that this questionnaire helps the treating physician in the assessing the severity and Quality of life and also in evaluating the outcome of treatment of sinonasal symptoms of COVID-19.

There are only few studies in literature that provides details on ENT symptoms in COVID-19 patients along with their quality of life using SNOT scores. Limitations of our study was it was more of a subjective analysis. Detailed ENT clinical examination was not conducted due to the risk of exposure and limited sources. A follow up study using the questionnaire would have helped in assessing the treatment modality.

## Conclusion

Based on our results, COVID-19 infected patients may just present with symptoms of Upper respiratory tract infections especially cough, loss of smell and taste without other significant complaints. There is a great need to develop a standard questionnaire which can deal with initial ENT symptoms among COVID-19 patients which helps to recognize and prevent further morbidity and mortality of the patients through early intervention. COVID-19 patients also suffer from significant psychological and sleep dysfunctions due to the ongoing sinonasal issues which needs to be addressed in a meticulous manner.

## Appendix

See Figs. 1 and 2.
